# Interaction between epilaryngeal and laryngeal adjustments in regulating vocal fold contact pressure

**DOI:** 10.1121/10.0003393

**Published:** 2021-02

**Authors:** Zhaoyan Zhang

**Affiliations:** Department of Head and Neck Surgery, University of California, Los Angeles, 31-24 Rehabilitation Center, 1000 Veteran Avenue, Los Angeles, California 90095-1794, USA zyzhang@ucla.edu

## Abstract

This study investigates the peak vocal fold contact pressure at different conditions of epilaryngeal narrowing and laryngeal adjustments. The results show that for a given subglottal pressure, the peak vocal fold contact pressure may increase or decrease with epilaryngeal narrowing, depending on a complex interaction between vocal fold vertical thickness, initial glottal angle, and subglottal pressure. However, epilaryngeal narrowing also significantly increases vocal efficiency so that for a target sound pressure level, the peak vocal fold contact pressure decreases with epilaryngeal narrowing. Overall, the peak vocal fold contact pressure and respiratory effort can be minimized by epilaryngeal narrowing, adopting a small initial glottal angle, and an intermediate vocal fold thickness.

## 1. Introduction

This study aims to better understand the effect of epilaryngeal narrowing on the peak contact pressure between the vocal folds during phonation, an important contributing factor to vocal fold injury. Epilaryngeal narrowing and other similar vocal tract adjustments have been widely used in vocal training and voice therapy (e.g., resonant voice therapy or semi-occluded phonation, Verdolini-Marston *et al.*, 1995; Verdolini *et al.*, 1998; Titze, 2006), with one important goal being to minimize vocal fold contact pressure. Titze (2006) showed in a computational study that epilaryngeal narrowing heightens interaction between the voice source and the vocal tract, and allows the supraglottal pressure to have a greater influence on the glottal flow, thus allowing the speaker to increase vocal intensity while minimizing glottal flow and the detrimental effects of vocal fold collision. Although a direct comparison between the output voice and vocal fold contact pressure was not made, Titze (2006) showed that epilaryngeal narrowing increases the ratio between the maximum flow declination rate (MFDR) and maximum area declination rate (MADR), a measure of vocal economy or output (voice)-cost (vocal fold collision) ratio. It was also pointed out that this effect would depend on the interaction between the degrees of epilaryngeal narrowing and vocal fold adduction.

This study investigates this interaction between epilaryngeal narrowing and laryngeal adjustments in controlling the peak vocal fold contact pressure between the vocal folds. In addition to varying the degree of initial glottal opening as in Titze (2006), we also consider changes in the vertical thickness of the vocal fold medial surface, which have been shown to have a large effect on the vocal fold closure pattern (Zhang, 2016a) and peak vocal fold contact pressure (Zhang, 2020). A three-dimensional, body-cover continuum model of voice production is used, which allows us to capture vocal fold collision dynamics with a better spatial resolution and directly evaluate the peak vocal fold contact pressure rather than the mean intraglottal pressure as investigated in Titze (2006). The specific goal is to determine the epilaryngeal and laryngeal conditions that produce the lowest peak vocal fold contact pressure in voice tasks either with a given constant subglottal pressure or targeting a specific sound pressure level (SPL).

## 2. Method

### 2.1 Computational model and simulation conditions

**Fig. 1.**
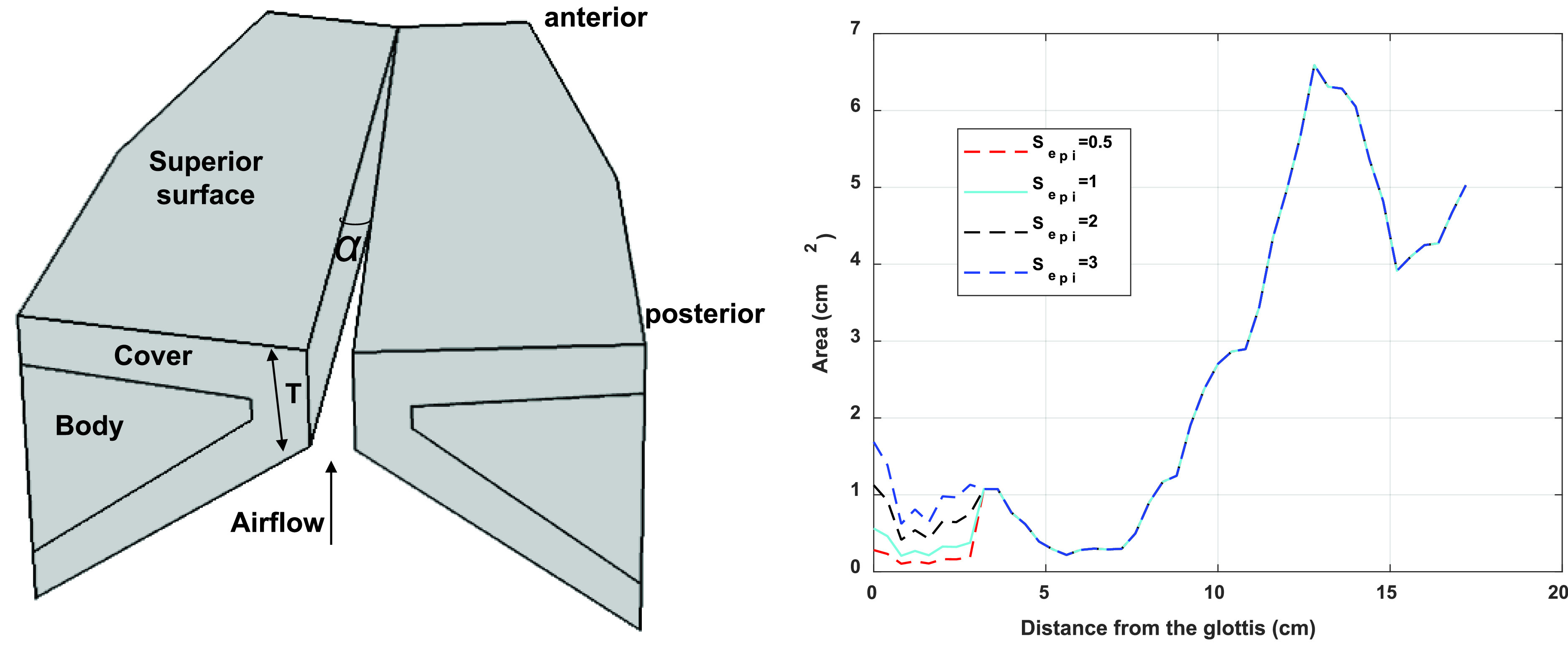
The three-dimensional vocal fold model and key geometric control parameters, including the vertical thickness of the medial surface *T* and the initial glottal angle *α* (left), and the vocal tract area functions with different epilaryngeal adjustments (right).

The same three-dimensional body-cover vocal fold model as in the study by Zhang (2020) is used in this study. The reader is referred to previous studies for details of the model (Zhang, 2017, 2019, 2020). The vocal fold model is parameterized by various geometric and mechanical properties of the vocal folds (Zhang, 2020). In this study, we consider parametric variations in the vocal fold medial surface vertical thickness *T* (1, 2, 3, and 4.5 mm) and the initial glottal angle *α* (0°, 1.6°, 4°, and 8°), which controls the degree of vocal fold approximation [Fig. 1(a)]. These two geometric controls have been shown to have a large impact on the peak vocal fold contact pressure in voice tasks with a target SPL (Zhang, 2020). The vocal fold model is fixed at the lateral surface and the two side surfaces at the anterior and posterior ends. Each vocal fold layer is modeled as a transversely isotropic, nearly incompressible, linear material with a plane of isotropy perpendicular to the anterior-posterior (AP) direction. The material control parameters for each vocal fold layer include the transverse Young's modulus *E_t_*, the AP Young's modulus *E_ap_*, the AP shear modulus *G_ap_*, and density. The effect of these mechanical properties on vocal fold contact pressure has been investigated in detail in the two studies by Zhang (2019, 2020). In this study, the body and cover layers have identical mechanical properties, with *E_t_*, *G_ap_*, and *E_ap_* set to 4 kPa, 10 kPa, and 40 kPa, respectively. The glottal flow is modeled as a one-dimensional quasi-steady glottal flow model taking into consideration viscous loss, as described in detail in Zhang (2017). Vocal fold contact occurs when portions of the vocal fold cross the glottal midline, in which case a penalty pressure along the medial-lateral direction into the vocal fold is applied to the contact surface of the vocal fold (Zhang, 2019). A large enough penalty pressure will ensure small penetration depth of the vocal folds crossing the glottal midline, and the corresponding penalty pressure will approximate the true contact pressure (Zhang, 2019).

The vocal tract is modeled as a one-dimensional waveguide (Story, 1995), and the cross-sectional area function corresponding to the /ɑ/ sound reported in Story *et al.* (1996; Fig. 6 on page 545) is used. The area function consists of 44 segments, each segment approximately 4 mm long, with a total vocal tract length of about 17.4 cm. Epilaryngeal manipulations are achieved by multiplying a scaling factor *S_epi_* uniformly to the cross-sectional areas of the eight segments closest to the glottis, which roughly covers the airway from the glottis to the level of the aryepiglottic folds (Fig. 1). Since the original area function already has a relatively small area in the epilaryngeal region, we implement one epilaryngeal narrowing manipulation and two expansion manipulations, with the scaling factor varying from 0.5, 1, 2, to 3. In the narrowing condition, the minimum epilaryngeal area is about 0.2 cm^2^, which is still within physiological range (Zhang *et al.*, 2019).

For each vocal fold and epilaryngeal condition, a half-second of voice production is simulated for 18 values of the subglottal pressure ranging from 50 Pa to 2.4 kPa, similar to Zhang (2020). From each simulation, the peak vocal fold contact pressure *P_c_* over the medial surface is calculated using the last 0.25 s of each simulation, by which time vocal fold vibration has either reached steady state or nearly steady state. Note that the peak contact pressure often occurs at different medial surface locations for different vocal fold conditions. The A-weighted SPL, which accounts for the frequency-dependence of loudness perception by the human ear, is extracted from the output acoustics as described in Zhang (2016a). The mean (Ag0) and peak-to-peak amplitude of the glottal opening area (Agamp) and the MADR are extracted from the glottal area waveform. From the glottal flow waveform, the mean glottal flow rate (Qmean), the peak-to-peak amplitude (Qamp), and the MFDR are extracted.

## 3 Results

### 3.1 Effect on glottal area and flow

**Fig. 2.**
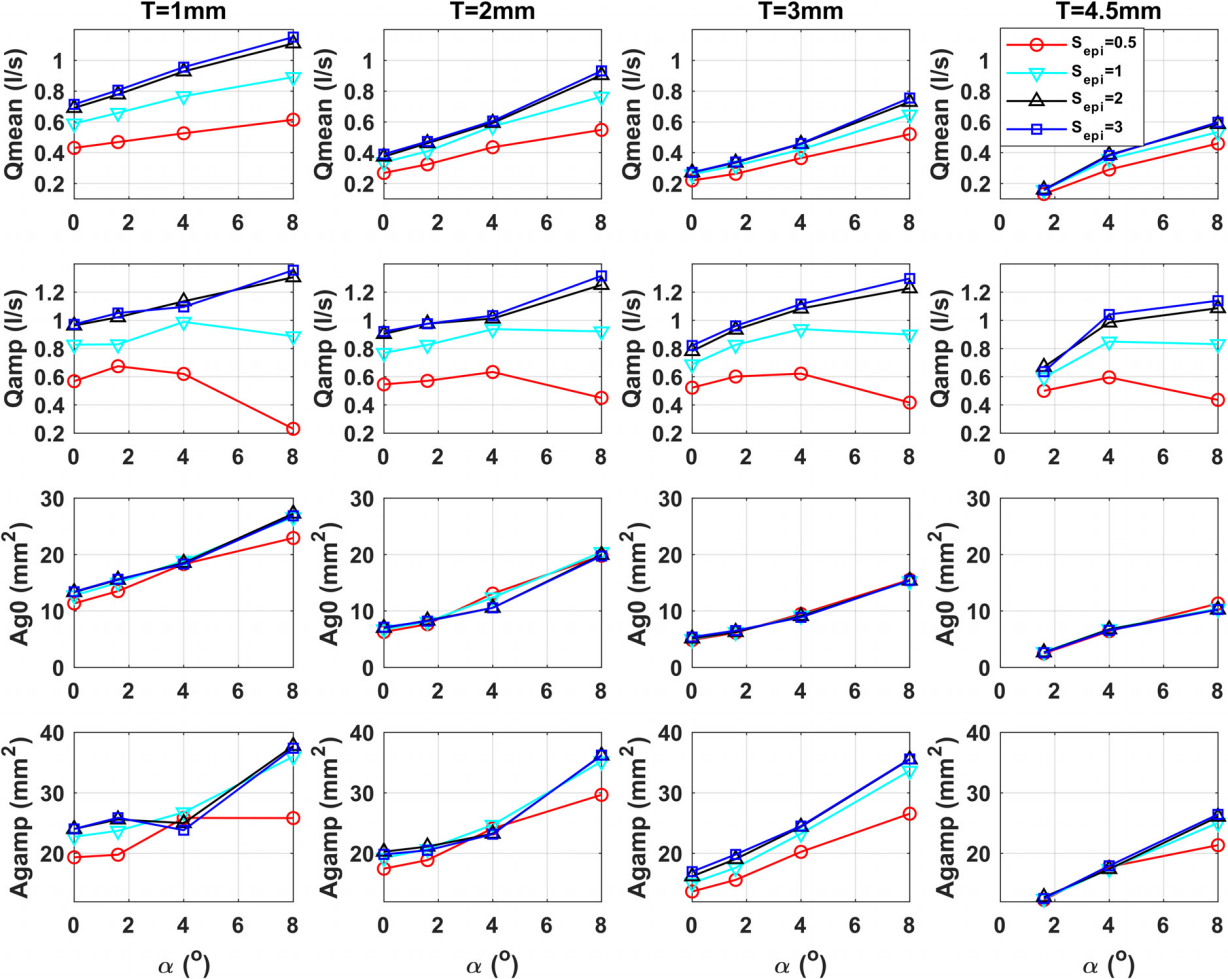
The means and peak-to-peak amplitudes of the glottal flow and area waveforms for different conditions of the initial glottal angle *α*, medial surface vertical thickness *T*, and degree of epilaryngeal narrowing *S_epi_*. Only data for conditions with sustained phonation are shown.

Figure 2 shows the effect of epilaryngeal adjustments on the means and peak-to-peak amplitudes of the glottal area and flow waveforms for different vocal fold conditions. The data are shown for a constant subglottal pressure, *Ps=2.2* kPa. This relatively high subglottal pressure is chosen because phonation is achieved in most conditions shown, which better illustrates the overall pattern. The general trends of variation are qualitatively similar for lower subglottal pressures.

As expected, epilaryngeal narrowing increases the overall airway resistance (Alipour *et al.*, 2007), both static and dynamic, and thus reduces both the mean and peak-to-peak amplitude of the glottal flow under a constant subglottal pressure, particularly for the narrowest epilaryngeal condition (*Sepi=0.5*). Figure 2 also shows that the effects of epilaryngeal narrowing on the glottal flow generally decrease with increasing vertical thickness or decreasing initial glottal angle, both of which increase the glottal resistance and presumably make the glottal flow less susceptible to epilaryngeal influence.

In contrast, while it is often hypothesized that increased back pressure from the vocal tract may slightly separate the vocal folds, the effect of epilaryngeal narrowing on the mean and peak-to-peak amplitude of the glottal area waveform is generally small (compare the bottom two rows with the first two rows in Fig. 2). For example, for *T=1* mm, extreme epilaryngeal narrowing almost reduces the mean glottal flow by half, whereas the mean glottal opening area remains almost the same. Similarly, although epilaryngeal narrowing generally reduces the peak-to-peak amplitude of the glottal area, the extent of this effect is much smaller compared with that on the peak-to-peak glottal flow amplitude.

### 3.2 Effect on SPL and peak vocal fold contact pressure

**Fig. 3.**
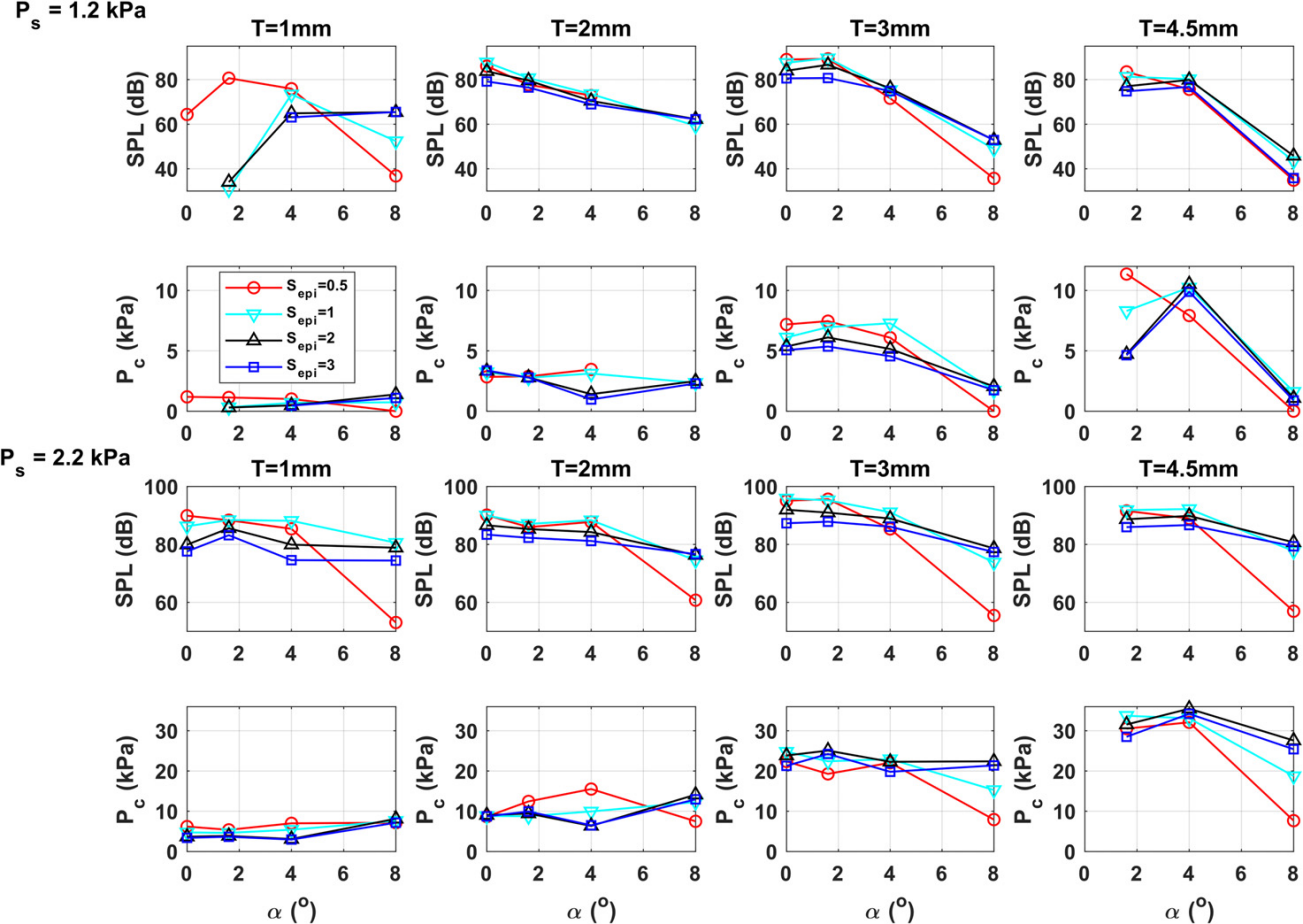
The SPL and peak contact pressure *P_c_* as a function of the initial glottal angle *α*, medial surface vertical thickness *T*, and degree of epilaryngeal narrowing *S_epi_*, for a subglottal pressure of 1.2 kPa (top two rows) and 2.2 kPa (bottom two rows).

Figure 3 shows the effect of epilaryngeal adjustments on the SPL and peak vocal fold contact pressure *P_c_* at different conditions of the initial glottal angle and vertical thickness for two subglottal pressures of 1.2 and 2.2 kPa. In general, epilaryngeal narrowing increases the SPL, except for the narrowest epilarynx condition at which the increase in SPL is small or even decreases slightly at some conditions, which is consistent with Titze (2002). In this study, vocal folds with an intermediate thickness (*T*=3 mm) are the most efficient with the highest SPL values.

For the peak contact pressure, the vertical thickness has the largest influence, with the peak contact pressure generally increasing with increasing vertical thickness, as in Zhang (2020). In comparison, the epilaryngeal effect on the peak contact pressure is smaller and inconsistent, and depends on a complex interaction between the vertical thickness, initial glottal angle, and subglottal pressure. For thin vocal folds or low to moderate subglottal pressures (e.g., *Ps*=1.2 kPa in the second row of Fig. 3), the peak contact pressure generally increases with increasing epilaryngeal narrowing under conditions of small initial glottal angles (*α* = 0–1.6°). However, for other conditions, the peak contact pressure generally increases first with epilaryngeal narrowing and then decreases with further epilaryngeal narrowing. The degree of epilaryngeal narrowing at which the peak contact pressure reaches maximum varies with the vertical thickness, initial glottal angle, and subglottal pressure. In general, the effect of the initial glottal angle on the peak contact pressure is small except for the largest initial glottal angle at which the peak contact pressure decreases rapidly, due to the high phonation threshold pressure and the gradual loss of vocal fold contact.

In this study, although both the MFDR and MADR (not shown) exhibit similar general trends as the peak contact pressure, none of the declination rate measures is a good indicator of the peak contact pressure. For example, for *T*=3 mm and *Ps*=1.2 kPa, epilaryngeal narrowing reduces the MADR but increases the peak contact pressure. This is probably because the peak contact pressure depends on the local vibratory pattern at the location of peak contact pressure, whereas both MFDR and MADR quantify global temporal characteristics of the glottal flow and vibration patterns.

### 3.3 Epilaryngeal effect when producing a target SPL level

**Fig. 4.**
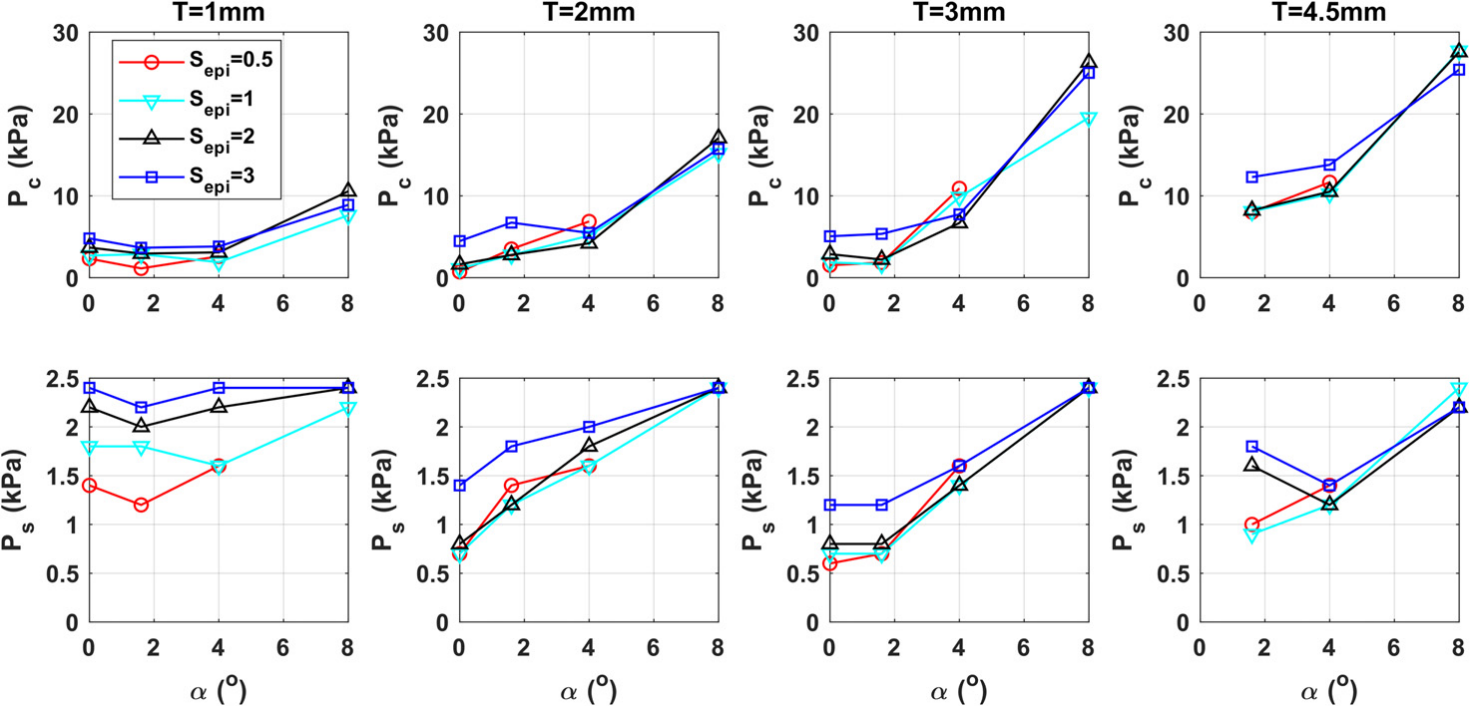
The peak contact pressure *P_c_* and subglottal pressure *P_s_* required to produce an 80 dB SPL for different conditions of the initial glottal angle *α*, medial surface vertical thickness *T*, and degree of epilaryngeal narrowing *S_epi_*.

Figure 4 shows the peak vocal fold contact pressure and the required subglottal pressure when producing a target SPL of 80 dB for different conditions of the initial glottal angle, vertical thickness, and epilaryngeal adjustments. The goal is to identify the laryngeal and epilaryngeal adjustments that can be made to minimize the peak contact pressure while producing an 80 dB SPL. In general, the peak contact pressure remains low for small initial glottal angles (0–4°), but increases rapidly when the initial glottal angle becomes larger. The peak contact pressure also shows a moderate increase with the vertical thickness, especially for thick vocal folds (*T* = 4.5 mm). These trends are consistent with the findings of Zhang (2020).

Figure 4 shows that epilaryngeal narrowing generally lowers the peak contact pressure for small initial glottal angles. This reduction is largely due to the improved voice production efficiency, which lowers the subglottal pressure required to produce an 80 dB SPL (lower panel in Figure 4). Because the subglottal pressure has the largest influence on the peak vocal fold contact pressure (Zhang, 2019), this reduced subglottal pressure requirement leads to an overall reduced peak contact pressure, despite the inconsistent effect of epilaryngeal narrowing on the peak contact pressure as shown in Fig. 3. In contrast, for large initial glottal angles, the effect of epilaryngeal narrowing gradually transitions to one that first increases then decreases the peak contact pressure with epilaryngeal narrowing, but the peak contact pressure remains high due to the very high subglottal pressure required to produce 80 dB at large initial glottal angles.

### 3.4 Comparison to previous studies

The results of this study are generally consistent with previous studies. For example, epilaryngeal narrowing in this study leads to a significant energy boost (as large as 15–20 dB) in the 2–3 kHz range in the voice spectra, similar to that in previous studies (Sundberg, 1974; Titze and Story, 1997). The reduction in the glottal flow rate with epilaryngeal narrowing is consistent with the observations in Titze (2002), Döllinger *et al.* (2006, 2012), and Samlan and Kreiman (2014). Samlan and Kreiman (2014) also showed the MFDR decreased with epilaryngeal narrowing, which is similar to the observations in our study for conditions of strong epilaryngeal narrowing. When normalized by the peak-to-peak flow amplitude and fundamental frequency, the normalized MFDR (the inverse of the normalized amplitude quotient) increases with epilaryngeal narrowing, particularly in thin vocal folds, indicating flow waveform skewing as expected from Rothenberg (1981). Our results also show the closed quotient generally decreases with increasing epilaryngeal narrowing.

## 4 Discussion and conclusions

This study shows that for a given constant subglottal pressure, while epilaryngeal narrowing consistently increases SPL, its effect on the peak vocal fold contact pressure is variable and depends on a complex interaction between the vertical thickness, initial glottal angle, and subglottal pressure. For thin vocal folds or low to moderate subglottal pressures, the peak contact pressure generally increases with increasing epilaryngeal narrowing at small initial glottal angles. For other conditions, the peak contact pressure first increases then decreases with epilaryngeal narrowing. Our results also show that epilaryngeal narrowing generally has a small effect on the mean and peak-to-peak amplitude of the glottal area waveform. This suggests that the observed changes in the peak contact pressure are in a large part due to subtle changes in vocal fold vibration dynamics induced by epilaryngeal narrowing (e.g., Döllinger *et al.*, 2012).

Despite this complex, variable effect on the peak contact pressure, the effect of epilaryngeal narrowing on the SPL is so dominant that epilaryngeal narrowing consistently leads to reduced peak contact pressure when targeting a specific SPL level, which supports the argument by Titze (2006) that epilaryngeal narrowing improves both vocal efficiency and vocal economy. The reduction in the peak contact pressure is largely due to the reduced requirement for subglottal pressure, which has been shown to have a much larger effect on vocal fold contact pressure than laryngeal or epilaryngeal adjustments. It is possible that other vocal tract adjustments, if they significantly increase vocal efficiency, may also reduce the peak contact pressure in voice tasks with a target SPL, which will be investigated in future studies.

Overall, our study shows that the peak vocal fold contact pressure can be minimized by epilaryngeal narrowing, adopting a small initial glottal angle (0–1.6°), and avoiding a very thick vocal fold configuration (*T*=1–3 mm in our study). In Fig. 4, such adjustments can decrease the peak contact pressure from, for example, 5 kPa to 0.7 kPa when producing an 80 dB SPL. In general, thin vocal folds allow this minimized peak contact pressure to be maintained in a relatively large range of initial glottal angles (0–4° for *T*=1 mm), but require slightly high subglottal pressure. In contrast, for an intermediate thickness (*T*=3 mm), the minimal peak contact pressure can be maintained in a smaller range, but the subglottal pressure requirement is slightly lower, which also reduces respiratory effort. This is the optimal vocal fold configuration that, when coupled with sufficient epilaryngeal narrowing, produces the desired vocal intensity with the least peak vocal fold contact pressure and respiratory effort. In humans, the initial glottal angle can be reduced by activating the lateral cricoarytenoid and thyroarytenoid muscles, while the vertical thickness can be reduced by activation of the cricothyroid muscles and increased by inferior-medial bulging of the medial surface due to the activation of the thyroarytenoid muscles (Zhang, 2016b). Since the laryngeal muscles that control the initial glottal angle and vertical thickness (i.e., the thyroarytenoid and lateral cricoarytenoid muscles), often co-activate in humans, it is possible that a barely-abducted glottal configuration, as often targeted in resonant voice therapy, would also help avoid a very thick vocal fold geometry, thus producing the optimal vocal configuration.

One limitation of this study is that the near field source-tract interaction in the epilaryngeal region may be oversimplified in our model. In particular, at extreme epilaryngeal narrowing, the false vocal folds may be in direct contact with the vocal folds and affect vocal fold vibration (Moisik and Esling, 2014), an effect that is not modeled in this study. Second, since only plane wave propagation in the vocal tract is considered and each vocal tract cross section is reduced to a single area value, epilaryngeal narrowing manipulations in this study do not differentiate between anterior-posterior and medial-lateral narrowing as often observed in humans, modeling of which may require high-dimensional acoustic models applied to three-dimensional imaging data. Also, only one vocal fold stiffness condition is considered in this study, although previous studies showed a small effect of vocal fold stiffness (Zhang, 2020). Finally, while the vocal tract model in this study includes multiple loss mechanisms, accurate modeling of these loss mechanisms is currently limited by the sparsity of experimental data, which may affect the results of this study, particularly for open glottal configurations in which epilaryngeal influence is strong. The results of this study thus need to be validated in future experiments.
